# Surface Plasmon Resonance-Enhanced CdS/FTO Heterojunction for Cu^2+^ Detection

**DOI:** 10.3390/s24123809

**Published:** 2024-06-12

**Authors:** Feng Chen, Mingfu Zhao, Bin Zhang, Minggang Zhao, Ye Ma

**Affiliations:** School of Material Science and Engineering, Ocean University of China, 238 Songling Road, Qingdao 266100, China

**Keywords:** Cu^2+^, heterojunction, gold nanoparticles, SPR, thermoelectric effect

## Abstract

Copper ion (Cu^2+^) pollution poses a serious threat to marine ecology and fisheries. However, the complexity of seawater and its interference factors make the online detection of Cu^2+^ quite challenging. To address this issue, we introduce the concept of the photo-assisted adjustment barrier effect into electrochemical detection, using it as a driving force to generate electrochemical responses. The Schottky barrier demonstrates a remarkable regulatory influence on the electrochemical response under photoexcitation, facilitating the response through Cu^2+^ adsorption. We developed a 4-MBA-AuNPs/CdS/FTO composite that serves as a sensitive platform for Cu^2+^ detection, achieving a detection limit of 70 nM. Notably, the photo-assisted adjustment of the barrier effect effectively counters the interference posed by ions in seawater, ensuring accurate detection. Furthermore, the sensor exhibits a promising recovery rate (99.62–104.9%) in real seawater samples, highlighting its practical applications. This innovative approach utilizing the photo-assisted adjustment barrier effect offers a promising path for developing electrochemical sensors that can withstand interference.

## 1. Introduction

Copper ions (Cu^2+^), as one of the important trace elements, can exert adverse effects on human health when accumulated excessively in the environment [1,2,3]. Therefore, the high-sensitivity and high-selectivity detection of Cu^2+^ in the environment is of great significance. Currently, there are various methods for detecting Cu^2+^, including fluorescence spectroscopy [4], mass spectrometry [5], molecular spectroscopy [6], and colorimetry [7]. Although these methods perform well in terms of accuracy and reliability, their high cost, complexity, and need for extensive sample pretreatment limit their application in trace detection. Electrochemical sensors have attracted widespread attention due to their simple operation, low cost, and rapid response to samples [8,9,10]. However, traditional electrochemical sensors are susceptible to interference when faced with complex environmental samples [11], thus requiring the development of new electrochemical sensors with an improved anti-interference performance. 

In opto-electrochemical sensors, advanced materials are crucial for improving sensor performance. Among these materials, cadmium sulfide (CdS) is of great interest due to its narrow bandgap, good conductivity, strong light absorption capacity, and appropriate band positions [12,13]. However, the insufficient surface active sites of CdS and the rapid recombination of photogenerated carriers can lead to photocorrosion under intense light irradiation, limiting its further application [14].

The Schottky junction structure, composed of metals and n-type semiconductor materials, features built-in electric fields and interface energy barriers. This unique heterojunction structure can effectively modulate electrochemical signals, promote charge transfer, and suppress carrier recombination, thereby significantly enhancing the photoelectric performance of materials [15,16,17]. Gold nanoparticles (Au NPs) exhibit excellent surface plasmon resonance (SPR) effects, outstanding biocompatibility, good conductivity, and a good catalytic performance [18,19,20]. Additionally, they possess an important mechanism known as photothermal–electric conversion (PHET) [21]. Under the PHET mechanism, plasmas excited by incident light rapidly decay into hot electron–hole pairs within a few to tens of femtoseconds, followed by the rapid transfer of hot electrons to adjacent semiconductors or molecules [22,23,24,25]. These characteristics make Au NPs highly favored in the field of electrochemical sensing [26].

In this study, we constructed a heterojunction-structured material that serves as the driving force for electrochemical signals in Cu^2+^ electrochemical sensing, exhibiting a stable current response. We selected fluorine-doped tin oxide (FTO) as the deposition substrate material for CdS and Au nanoparticles (Au NPs). Through Au-S bond coupling, 4-mercaptobenzoic acid (4-MBA) binds to Au NPs, wherein the carboxyl groups (-COOH) in the 4-MBA molecules can chelate with Cu^2+^, forming a 4-MBA-Cu^2+^-4-MBA complex [27]. The specificity of Au NPs containing the 4-MBA ligand for Cu^2+^ adsorption enhances the sensor’s resistance to interference. Additionally, the surface plasmon resonance (SPR) effect of the Au NPs enhances the photoelectric current response of the 4-MBA-AuNPs/CdS/FTO material. Under illumination, high-energy electrons excited on the surface of the Au NPs and photogenerated electrons from the CdS are simultaneously injected into the conduction band of CdS, thereby increasing the sensitivity of this electrochemical sensor. By fully exploiting the Schottky barrier and the hot-electron effect excited by Au NPs and ingeniously coordinating the relationship between the barrier and illumination, we successfully developed a novel electrochemical sensor with excellent stability.

## 2. Materials and Methods

### 2.1. Chemicals and Characterizations

Trisodium citrate, hydroxylamine hydrochloride, 4-mercaptobenzoic acid, 4-mercaptopyridine, tetrabutylammonium tetraphenylborate, hydrochloric acid, lithium chloride, 1,2-dichloroethane, sodium hydroxide, mercuric nitrate, nickel nitrate, copper nitrate, and calcium nitrate were purchased from Aladdin (Shanghai) Holding Group. Sodium chloride, ferrous chloride, cadmium chloride, potassium chloride, and sodium fluoride were purchased from Macklin (Shanghai) Holding Group. Ethanol, sodium thiosulfate, potassium ferrocyanide, ethylenediaminetetraacetic acid, disodium phosphate, and monosodium phosphate were purchased from China National Pharmaceutical Group. Ultra-pure water (UP) with a resistivity of 18.25 MΩ·cm was used in the experiments.

The morphology of the samples was characterized using a scanning electron microscope (SEM) (ZEISS Sigma 300, ZEISS, Oberkochen, Germany) and a transmission electron microscope (TEM) (JEOL JEM, JEOL Ltd., Akishima, Japan). Fourier-transform infrared spectroscopy measurements were conducted using a Thermo Scientific Nicolet iS20 Fourier-transform infrared spectrometer (Thermo Fisher Scientific, Waltham, MA, USA). The pH value of the solutions was determined using a PHS-3D pH meter from Shanghai Precision Scientific Instrument (Shanghai, China). The composition of the samples was analyzed using an X-ray diffractometer (Ultima IV, Rigaku Corporation, Akishima City, Tokyo, Japan). The composition of each element in the samples was analyzed using an energy dispersive spectrometer (EDS) (ZEISS Sigma 300) and an X-ray photoelectron spectrometer (XPS) (Thermo Scientific, Waltham, MA, USA). UV-visible absorption spectra were recorded using a UV-8000 spectrophotometer. Samples were subjected to photocatalytic treatment using a photocatalytic apparatus (PL-X5000-FH) from Beijing Purkinje General Science & Technology (Beijing, China), equipped with a simulated sunlight xenon lamp light source (PL-X5000, Aquascape, Hong Kong). Electrochemical signals were measured using an electrochemical workstation (CHI660E, CH Instruments, Inc., Shanghai, China).

### 2.2. Preparation of 4-MBA-AuNPs/CdS/FTO Composites

FTO conductive glass pretreatment: Firstly, a piece of FTO conductive glass with dimensions of 1 cm² was placed into an ultrasonic cleaner. It was sequentially cleaned with acetone, ethanol, and ultra-pure water for 15 min, each using ultrasonic agitation. Finally, the cleaned FTO conductive glass was dried in an oven.

Deposition of CdS: In a three-electrode system, FTO conductive glass was selected as the working electrode, platinum wire as the counter electrode, and a saturated calomel electrode as the reference electrode. The electrodes were first immersed in a phosphate-buffered solution (PBS) containing 0.32 mM Na_2_S_2_O_3_·5H_2_O, 0.32 mM EDTA, and 0.64 mM CdCl_2_. Electrochemical deposition was then carried out at a constant voltage of −1.06 V for 600 s to obtain CdS/FTO.

Preparation of 4-MBA-Au NPs: Gold nanoparticles (Au NPs) with a diameter of 40 nm in spherical shape were prepared using seed growth and reduction methods. Firstly, 0.5 mL of 5% (wt) HAuCl4·3H_2_O aqueous solution was thoroughly mixed with 400 mL of ultra-pure (UP) water in a three-neck flask. Subsequently, the mixture was heated and stirred using a magnetic stirrer until boiling and then allowed to boil for 30 min under condensation. Then, 8 mL of 1% (wt) sodium citrate solution was added to the flask, and the reaction continued for another 30 min. After natural cooling, Au NPs with a particle size of 16 nm were obtained. Next, the 16 nm Au NPs were mixed and stirred with UP water for 10 min, followed by the addition of NH_2_OH solution and stirring for 5 min. Then, 520 μL of 5% (wt) HAuCl_4_·3H_2_O aqueous solution was added, and the reaction proceeded for 15 min to obtain 40 nm Au NPs. The pH of the prepared citrate-functionalized 40 nm Au NPs solution was adjusted to pH = 9.0 by adding NaOH or HCl, and then 4-mercaptobenzoic acid (4-MBA) ligand was added to a final concentration of 2 μM. After incubating for 15 min, the solution was centrifuged at 2000 RCF for 45 min to remove free ligands in the supernatant, and the precipitated Au NPs at the bottom were redispersed in UP water.

Preparation of 4-MBA-Au NPs film: Gold nanoparticles (Au NPs) containing LiCl and dichloroethane (DCE) organic phase containing tetrabutylammonium tetraphenylborate (TBATPB) were injected into the electrolytic cell. When a negative potential relative to the point of zero charge (P.Z.C.) was applied to the aqueous phase, negatively charged Au NPs gradually migrated to the liquid–liquid interface (LLI), where the Au NPs were assembled into an NP array with strong surface plasmon resonance (SPR) coupling.

Preparation of 4-MBA-AuNPs/CdS/FTO: The assembled 4-MBA-AuNPs film at the liquid–liquid interface (LLI) was transferred to the dried CdS/FTO substrate. The attached solvents on the final 4-MBA-AuNPs/CdS/FTO were allowed to evaporate in the oven.

### 2.3. Electrochemical Signal Test

Electrochemical tests were conducted on an electrochemical workstation. In the preparation of 4-MBA-Au NPs, a four-electrode working system was employed, with Ag/AgCl electrodes chosen as the reference and counter electrodes, and a platinum (Pt) ring electrode used as the working electrode. For Cu^2+^ detection, a traditional three-electrode system was utilized, with 4-MBA-AuNPs/CdS/FTO composite material as the working electrode, a Pt wire electrode as the counter electrode, and Ag/AgCl electrode as the reference electrode. A PL-X500D xenon lamp was used as a simulated solar light source (Aquascape, Hong Kong). The electrochemical detection of Cu^2+^ was conducted in 1 mM pH 9.0 phosphate-buffered saline (PBS) solution. Differential pulse voltammetry (DPV) spectra were recorded with a scanning rate of 50 mV/s.

## 3. Results and Discussion

### 3.1. Material Characterization

**Figure 1:**
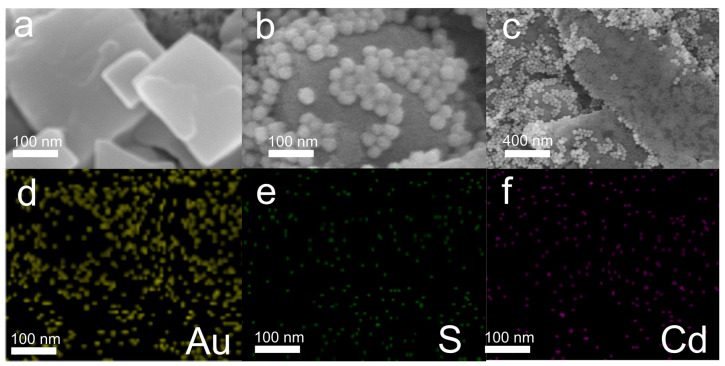
(**a**) SEM image of CdS/FTO. (**b**) SEM image of 4-MBA-AuNPs/CdS/FTO. (**c**) Low-magnification SEM image of 4-MBA-AuNPs/CdS/FTO. And EDS mapping images of Au (**d**), S (**e**), and Cd (**f**) of 4-MBA-AuNPs/CdS/FTO nanocomposite materials.

SEM images (Figure 1a–c) confirmed the successful preparation of 4-MBA-AuNPs/CdS/FTO, showing spherical 4-MBA-Au NPs decorating the surface of the CdS/FTO. The results of an EDS elemental mapping analysis (Figure 1d–f) display the distributions of the Au, Cd, and S elements. Figure S1 shows the XRD spectrum of the 4-MBA-AuNPs/CdS/FTO composite material, where the peaks observed at 37.9°, 44.4°, and 78.2° correspond to the (111), (200), and (311) crystal planes of Au (JCPDS 04-0784) [28], respectively. The peaks observed at 26.5°, 33.7°, 43.4°, 51.9°, and 54.5° correspond to the (111), (200), (220), (311), and (222) crystal planes of CdS (JCPDS 10-0454) [29], respectively. Additionally, the peaks observed at 33.7°, 37.9°, 51.9°, 54.5°, 61.6°, and 65.6° correspond to the (101), (200), (211), (220), (310), and (301) crystal planes of SnO_2_ (JCPDS 71-0652) [30], respectively. No other impurity peaks were observed, confirming the successful preparation of the 4-MBA-AuNPs/CdS/FTO nanocomposite material.

XPS characterization further validated the elemental valence states and chemical composition of 4-MBA-AuNPs/CdS/FTO. In Figure S2a, the full spectrum shows the presence of the constituent elements Au, Cd, and S, confirming the successful synthesis of the composite material. Figure S2b displays the high-resolution binding energies of Au 4f_7/2_ and Au 4f_5/2_ at 83.3 eV and 87.1 eV, respectively, indicating the presence of gold [31]. The Cd 3d XPS spectrum shown in Figure S3c forms two peaks at binding energies of 405.3 eV and 412.1 eV, corresponding to the Cd 3d_5/2_ and Cd 3d_3/2_ of Cd^2+^ in CdS, respectively [32]. Figure S2d shows the S 2P XPS spectrum with two peaks at binding energies of 160.7 eV and 162.1 eV, corresponding to S 2P_3/2_ and S 2P_1/2_, respectively, indicating the presence of sulfur in the form of S^2−^ [33].

### 3.2. Working Principle of Electrochemical Detection

**Figure 2:**
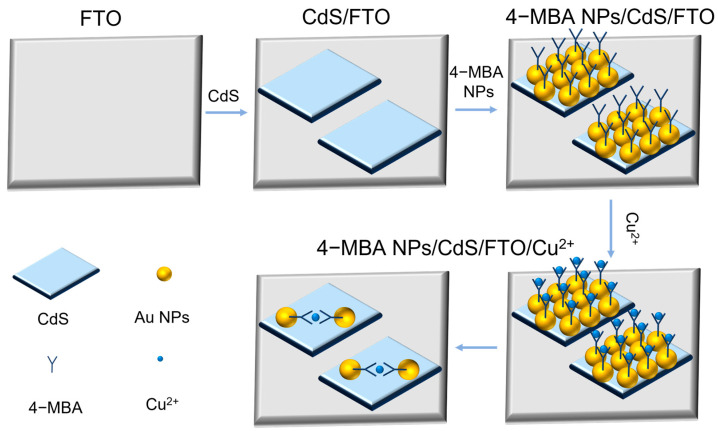
Schematic of the synthesis of 4-MBA-AuNPs/CdS/FTO nanocomposites.

**Figure 3:**
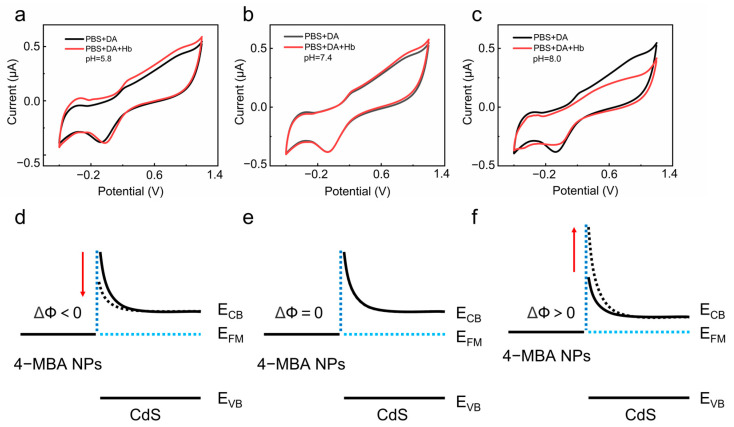
Adding HB with different charge states to DA solution and recording the corresponding CV curves and Schottky barrier changes. (**a**,**d**) Positively charged HB. (**b**,**e**) Uncharged HB. (**c**,**f**) Negatively charged HB.

The working principle of the sensing platform is illustrated in Figure 2. In utilizing the electrostatic induction method in dopamine (DA), the influence of the Schottky barrier at the interface of 4-MBA-Au NPs/CdS on the electrochemical response was investigated. In this process, DA serves a key role as the redox mediator. The electrostatic induction phenomenon is successfully induced by adding hemoglobin (HB) to the DA solution. Additionally, the isoelectric point of HB is at pH = 7.4, indicating that under different pH environments (pH = 8.0, 7.4, 5.8), HB will exhibit negative, neutral, and positive charges. Figure 3 clearly demonstrates the cyclic voltammetry curves after adding HB to the DA solution at different pH values. In observing these curves, it can be noted that charged HB in the buffer solution induces electrostatic induction on the 4-MBA-AuNPs/CdS/FTO composite material, resulting in the induction of opposite charges. This electrostatic induction directly affects the Schottky barrier, thereby driving the response of the electrochemical signal.

At pH = 5.8, HB carries a positive charge. When added to the buffer solution, it increases the current (Figure 3a). This is because the positively charged HB induces electron accumulation on the CdS conduction band, leading to decreases in the conduction band and the interface barrier (ΔΦ < 0) (Figure 3d), thereby enhancing the response of the electrochemical signal. At pH = 7.4, HB is neutral, and adding it to the buffer solution causes little change in the current (Figure 3b). This is because the neutral charge does not alter the Schottky barrier (ΔΦ = 0) (Figure 3e), resulting in no change in the electrochemical signal response. At pH = 8.0, HB carries a negative charge. Adding it to the buffer solution decreases the current (Figure 3c). This is because the negatively charged HB causes the CdS conduction band to rise, leading to an increase in the interface barrier (ΔΦ > 0) (Figure 3f), resulting in a decrease in the response of the electrochemical signal.

**Figure 4:**
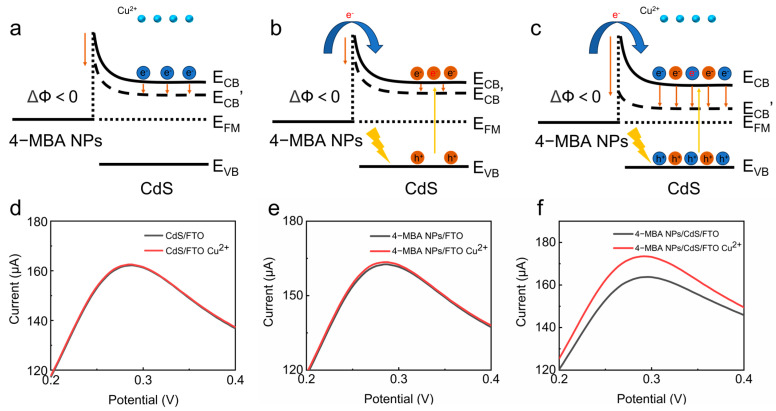
Changes in the potential barrier for (**a**) adsorbed Cu^2+^ in the dark, (**b**) unabsorbed Cu^2+^ in the light, and (**c**) adsorbed Cu^2+^ in the light. DPV curves of (**d**) CdS/FTO, (**e**) 4-MBA-Au NPs/FTO, and (**f**) 4-MBA-AuNPs/CdS/FTO after the addition of 1000 nM Cu^2+^ at pH = 9.0.

As shown in Figure 4, the Schottky barrier formed at the interface between the CdS and 4-MBA-Au NPs in the 4-MBA-AuNPs/CdS/FTO nanocomposite material causes a bending of the CdS band, creating an interface barrier that hinders charge transfer from CdS to 4-MBA-Au NPs. Due to the abundance of -COOH on the surface of the 4-MBA-Au NPs, the selective adsorption of Cu^2+^ occurs. When positively charged Cu^2+^ is adsorbed onto the surfaces of the 4-MBA-Au NPs, forming a 4-MBA-Cu^2+^-4-MBA chelate, the positive charge of Cu^2+^ induces free electrons on the surface of CdS through electrostatic induction. The conduction band of CdS decreases due to electron accumulation, resulting in a decrease in the interface barrier, thereby increasing the electrochemical response. As the concentration of Cu^2+^ adsorbed on the surfaces of the 4-MBA-Au NPs increases, the accumulation of electrons on the surface of the CdS also increases, further reducing the barrier, thereby increasing the electrochemical response further (Figure 4a). In establishing a linear relationship between the current change and the concentration of Cu^2+^, the detection of Cu^2+^ is achieved. After applying certain illumination, a large number of electrons are excited from the valence band to the conduction band of CdS. Meanwhile, due to the surface plasmon resonance (SPR) effect and the photothermal–electric (PHET) mechanism, high-energy hot electrons are generated on the surfaces of the 4-MBA-Au NPs. These electrons cross the barrier and transfer to the conduction band of CdS, causing a further reduction in the CdS conduction band, thereby increasing the change in the Schottky barrier (Figure 4b). When Cu^2+^ is adsorbed, CdS accumulates more electrons in the conduction band, leading to a more significant change in the barrier, resulting in a more pronounced electrochemical response (Figure 4c).

### 3.3. Electrochemical Testing

To validate the role of the Schottky barrier, we conducted a differential pulse voltammetry (DPV) curve analysis by separately adding the same concentration of Cu^2+^ into the CdS/FTO, 4-MBA-Au NPs/FTO, and 4-MBA-AuNPs/CdS/FTO. The results show that compared to the FTO surface with the single-phase deposition of CdS (Figure 4d) and 4-MBA-Au NPs (Figure 4e), the current significantly increased and tended to stabilize on the surface of the CdS/FTO after introducing 4-MBA-Au NPs (Figure 4f). This indicates that the Schottky barrier drives the electrochemical current response.

**Figure 5:**
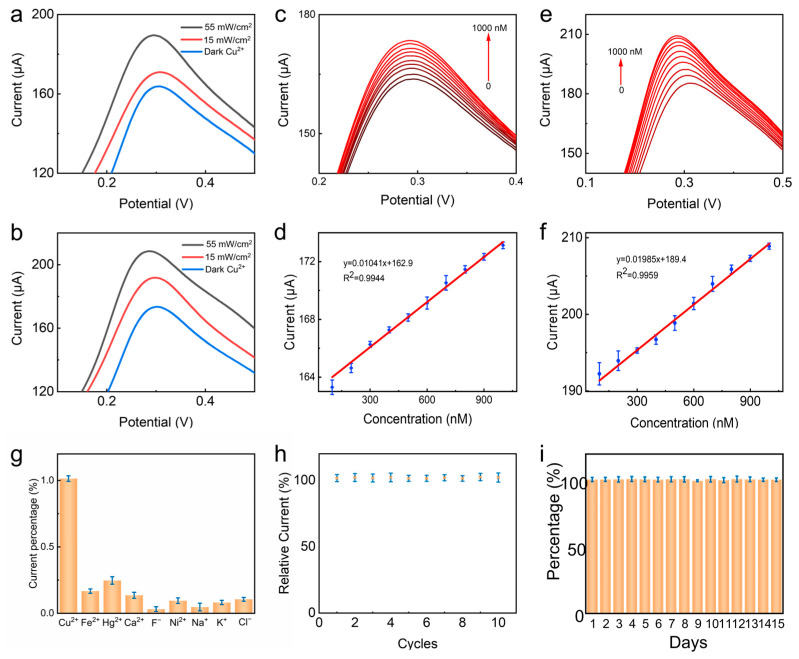
(**a**) DPV curves of 4-MBA-AuNPs/CdS/FTO composites under dark, 15 mW/cm^2^, and 55 mW/cm^2^ xenon lamps without adsorbed Cu^2+^. (**b**) DPV curves of 4-MBA-AuNPs/CdS/FTO composites under dark, 15 mW/cm^2^, and 55 mW/cm^2^ xenon lamps for the adsorption of 1000 nM Cu^2+^. (**c**) Corresponding changes in the DPV curve after the addition of different concentrations of Cu2+ to a PBS solution at pH = 9.0 in the dark. (**d**) Calibration curve of Cu^2+^ concentration versus peak current under dark conditions. (**e**) Corresponding changes in DPV curves after adding different concentrations of Cu^2+^ to PBS solution at pH = 9.0 under 55 mW/cm^2^ light conditions. (**f**) Calibration curve of Cu^2+^ concentration versus peak current under light conditions. (**g**) Electrochemical current response of 4-MBA-AuNPs/CdS/FTO composites to different ions at the same concentration. (**h**) DPV response of the 4-MBA-AuNPs/CdS/FTO composite for 10 cycles. (**i**) The DPV response of the composite was measured for 15 consecutive days.

Next, we conducted light irradiation contrast experiments to verify the influence of light on the material. Figure 5 shows the changes in electrochemical response current of the 4-MBA-AuNPs/CdS/FTO composite material before and after the adsorption of Cu^2+^ under dark, 15 mW/cm^2^ xenon lamp, and 55 mW/cm^2^ xenon lamp conditions. The results indicate that with increasing light intensity, the electrochemical response current signal also increases accordingly. In particular, the current under 55 mW/cm^2^ xenon lamp conditions significantly increases compared to the dark conditions (Figure 5a). After the adsorption of Cu^2+^, as the light intensity increases, the change in the barrier becomes more significant, leading to a larger variation in the electrochemical response current. Therefore, light can modulate the height of the barrier, thereby enhancing the sensing performance (Figure 5b).

Based on the experimental results described above, we first conducted differential pulse voltammetry (DPV) testing on the 4-MBA-AuNPs/CdS/FTO composite material under dark conditions. As shown in Figure 5c, with the addition of Cu^2+^, the oxidation peak exhibited a noticeable increase. Particularly with an addition of Cu^2+^ within the range of 0 to 1000 nM , the electrochemical response current displayed a proportional growth trend with the Cu^2+^ concentration, indicating a significant influence of Cu^2+^ concentration on the electrochemical response. Further analysis through fitting, as shown in Figure 5d, revealed a linear relationship between the Cu^2+^ concentration (x) and the response current (y), with a fitting equation of y = 0.01041x + 162.9, an R^2^ of 0.9944, and a detection limit of 132 nM.

Under illumination conditions, the utilization of the photothermal–electrochemical (PHET) mechanism enables the effective enhancement of electrochemical sensing through the generation of hot electrons on the surfaces of Au NPs. To further investigate the influence of illumination, we conducted photoelectrochemical testing on the composite material using a 55 mW/cm^2^ simulated xenon lamp. As shown in Figure 5e, after the addition of Cu^2+^ in the range of 0 to 1000 nM, the change in the electrochemical response current became more significant. This is attributed to the surface plasmon resonance (SPR) effect of Au NPs, which enhances the absorption of incident light, leading to the injection of hot electrons into the conduction band of CdS, resulting in electron accumulation and, consequently, a decrease in the barrier height, leading to an increase in current. As depicted in Figure 5f, a fitting analysis was performed on the Cu^2+^ concentration (x) and the response current (y), yielding a linear equation of y = 0.01985x + 189.4, an R^2^ of 0.9959, and a limit of detection (LOD) of 70 nM. Compared to other sensors, the proposed sensor’s performance is outstanding (Table S1). Indeed, there also exist sensors capable of obtaining lower detection limits [34,35,36,37], but the main aim of this work was to propose a novel sensing prototype combining the tunable Schottky barrier and PHET effect.

### 3.4. Specificity and Long-Term Stability

The selectivity and anti-interference properties of the prepared material were evaluated by detecting the response to other ions. As shown in Figure 5g, the response curves of the 4-MBA-AuNPs/CdS/FTO composite material to the same concentrations of Cu^2+^, Fe^2+^, Hg^2+^, Ca^2+^, F^−^, Ni^2+^, Na^+^, K^+^, and C^l−^ exhibit distinct characteristics. Clearly, the sensor’s response to Cu^2+^ is significantly higher than that to other ions, indicating its excellent selectivity and anti-interference properties.

To verify the stability of the composite material, we conducted 15 cycles of DPV testing and DPV testing for 15 days in PBS buffer solution at pH 7.0. As observed from Figure 5h, within the first five cycles of testing, the material exhibited good stability and repeatability. This indicates that under specific environmental conditions, the material possesses durability and reliability, maintaining a stable performance. Additionally, Figure 5i shows that the material exhibits good long-term stability during cyclic DPV testing, indicating consistent performance over time.

### 3.5. Detection of Real Water Samples

To validate the potential of the electrochemical sensor for detecting Cu^2+^ in real samples, we tested seawater and tap water samples. The performance of the sensor in DPV mode is shown in Table 1, with average recovery rates ranging from 99.62% to 104.9%. This indicates that the sensor has excellent efficacy for determining Cu^2+^ concentrations in real samples.

## 4. Conclusions

We successfully developed a novel electrochemical sensor based on 4-MBA-AuNPs/CdS/FTO nanocomposites for the sensitive detection of Cu^2+^. The integration of Au NPs and CdS nanocrystals into an FTO conductive glass substrate facilitated the formation of a Schottky junction, which effectively modulates the electrochemical signals. The specific adsorption of Cu^2+^ by the 4-MBA ligand on the surfaces of the Au NPs enhances the sensor’s anti-interference properties. The heterostructure of 4-MBA-AuNPs/CdS/FTO, driven by electrochemical signals, demonstrates stable current responses. Moreover, the surface plasmon resonance (SPR) effect of Au NPs enhances the photoelectric current response of the sensor, especially under light exposure, further improving its sensitivity. Through a thorough utilization of the Schottky barrier and the hot-electron effect induced by Au NPs, combined with the coordination between the barrier and light exposure, we successfully developed a new electrochemical sensor with excellent stability. This sensor exhibits promising potential for practical applications in the detection of Cu^2+^, demonstrating its reliability and practicality even in complex samples such as sea water.

## Figures and Tables

**Table 1:** Measuring real-life samples using DPV.

Sample	Spiked (nM)	Found (nM)	Recovery (%)	RSD (%)
Tap water	300	298.9 ± 23.3	99.6	1.5
	600	628.6 ± 13.8	104.8	2.5
	800	804.7 ± 11.7	100.6	0.4
Sea water	300	314.9 ± 2.1	104.9	0.8
	600	622.2 ± 6.1	103.7	0.6
	800	831.3 ± 19.9	103.9	1.6

## Data Availability

Data will be made available upon request.

## Supplementary Materials

The following supporting information can be downloaded at: https://www.mdpi.com/article/10.3390/s24123809/s1, Figure S1. XRD spectra of 4-MBA-AuNPs/CdS/FTO composites. Figure S2. (a) Full XPS spectrum of 4-MBA-AuNPs/CdS/FTO nanocomposites. (b) XPS spectra of Au 4f orbitals. (c) XPS spectra of Cd 3d orbitals. (d) XPS spectra of S 2P orbitals. Table S1. Performance comparison of 4-MBA-AuNPs/CdS/FTO composites with other sensors. References [34,35,36,37,38,39,40,41,42,43,44,45] are cited in the Supplementary Materials.
