# Supplementary material for: Surface Plasmon Resonance-Enhanced CdS/FTO Heterojunction for Cu2+ Detection
Source: Sensors (Basel). 2024 Jun 12;24(12):3809. doi: 10.3390/s24123809 (PMC11207611; doi:10.3390/s24123809)
Supplement: Supplementary file 1 [file sensors-24-03809-s001.zip › sensors-3007580-supplementary.pdf]

Supporting Information

Surface Plasmon Resonance-Enhanced CdS/FTO Heterojunction for Cu^{2+} Detection

Feng Chen †, Mingfu Zhao †, Bin Zhang, Minggang Zhao * and Ye Ma *

School of Material Science and Engineering, Ocean University of China, 238 Songling Rd, Qingdao 266100, China

* Correspondence: zhaomg@ouc.edu.cn (M.Z.); maye@ouc.edu.cn (Y.M.)

† These authors contributed equally to this work.

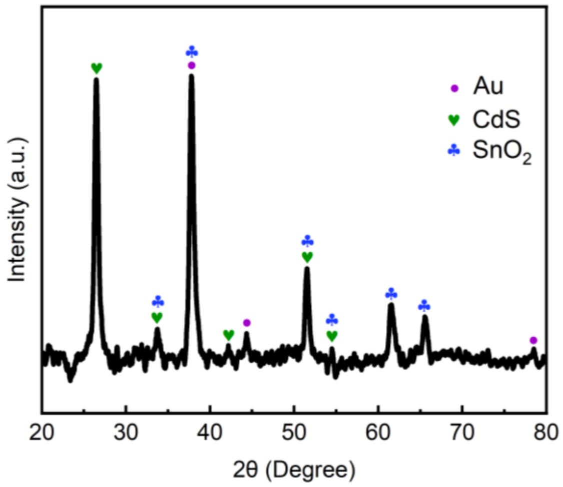

Figure S1. XRD spectra of 4-MBA-AuNPs/CdS/FTO composites.

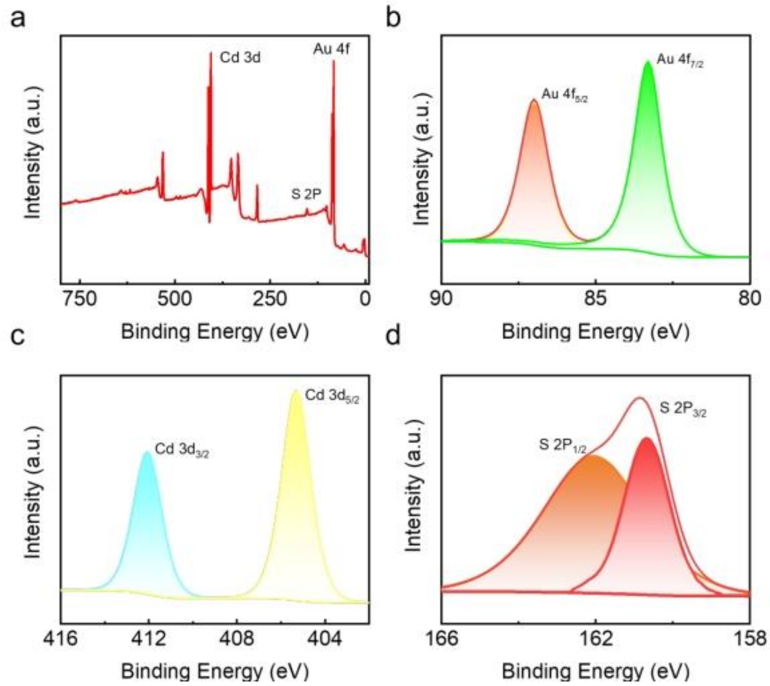

Figure S2. (a) Full XPS spectrum of 4-MBA-AuNPs/CdS/FTO nanocomposites. (b) XPS spectra of Au 4f orbitals. (c) XPS spectra of Cd 3d orbitals. (d) XPS spectra of S 2P orbitals.

Table S1 Performance comparison of 4-MBA-AuNPs/CdS/FTO composites with other sensors.

Sensor	Method	Linear range (μM)	LOD (nM)	Ref.
$NaYF_4$: Yb^{3+}/Er^{3+}-RBH	Fluorescence	1–10	1000	[1]
DST@DNA	Fluorescence	0.5–10	290	[2]
coumarin 334	Fluorescence	0–0.8	87	[3]
ketodipicolymine	Fluorescence	0.3–50	100	[4]
DNA zymes	Chemiluminescence	0.625–15	290	[5]
Click chemistry	Colorimetric assay	0.5–10	250	[6]
CQDS	Fluorescence	0-15	226	[7]
CMH-GA-CDs	Fluorescence	0-10	210	[8]
S,N-CQDs	Chemiluminescence	0.15–7.8	31.5	[9]
VMSF/ITO	Electrochemistry	0.1–30	20	[10]
Gold nanostars	Chemiluminescence	0.002–9.0	0.9	[11]

Au@Ag nanoparticles	Electrochemistry	0.001–100	0.3	[12]
4-MBA-AuNPs/CdS/FTO	With Light	0-10	70	This work
	Dark	0-10	132	

References

1. Zhang J., Li B., Zhang L., Jiang H., An optical sensor for Cu(ii) detection with upconverting luminescent nanoparticles as an excitation source. *Chem. Commun.* **2012**, *48*, 4860-4862.
2. Shen Q., Tang S., Li W., Nie Z., Liu Z., Huang Y., Yao S., A novel DNA-templated click chemistry strategy for fluorescent detection of copper(ii) ions. *Chem. Commun.* **2012**, *48*, 281-283.
3. Kim M.H., Jang H.H., Yi S., Chang S.-K., Han M.S., Coumarin-derivative-based off–on catalytic chemodosimeter for Cu2+ ions. *Chem. Commun.* **2009**, 4838-4840.
4. Cho S.W., Rao A.S., Bhunia S., Reo Y.J., Singha S., Ahn K.H., Ratiometric fluorescence detection of Cu(II) with a keto-dipicolylamine ligand: A mechanistic implication. *Sens. Actuators B Chem.* **2019**, *279*, 204-212.
5. Wang Y., Yang F., Yang X., Label-free colorimetric biosensing of copper(II) ions with unimolecular self-cleaving deoxyribozymes and unmodified gold nanoparticle probes. *Nanotechnology* **2010**, *21*, 205502.
6. Shen Q., Li W., Tang S., Hu Y., Nie Z., Huang Y., Yao S., A simple "clickable" biosensor for colorimetric detection of copper(II) ions based on unmodified gold nanoparticles. *Biosens. Bioelectron.* **2013**, *41*, 663-668.
7. Wang Y., Zhang C., Chen X., Yang B., Yang L., Jiang C., Zhang Z., Ratiometric fluorescent paper sensor utilizing hybrid carbon dots–quantum dots for the visual determination of copper ions. *Nanoscale* **2016**, *8*, 5977-5984.
8. Yan F., Bai Z., Chen Y., Zu F., Li X., Xu J., Chen L., Ratiometric fluorescent detection of copper ions using coumarin-functionalized carbon dots based on FRET. *Sens. Actuators B Chem.* **2018**, *275*, 86-94.
9. Amjadi M., Manzoori J.L., Hallaj T., Azizi N., Sulfur and nitrogen co-doped carbon quantum dots as the chemiluminescence probe for detection of Cu2+ ions. *J. Lumin.* **2017**, *182*, 246-251.
10. Cheng B., Zhou L., Lu L., Liu J., Dong X., Xi F., Chen P., Simultaneous label-free and pretreatment-free detection of heavy metal ions in complex samples using electrodes decorated with vertically ordered silica nanochannels. *Sens. Actuators B Chem.* **2018**, *259*, 364-371.
11. Amjadi M., Abolghasemi-Fakhri Z., Gold nanostar-enhanced chemiluminescence probe for highly sensitive detection of Cu(II) ions. *Sens. Actuators B Chem.* **2018**, *257*, 629-634.
12. Wu T., Xu T., Ma Z., Sensitive electrochemical detection of copper ions based on the copper(ii) ion assisted etching of Au@Ag nanoparticles. *Analyst* **2015**, *140*, 8041-8047.
